# Study on Tungsten Metallization and Interfacial Bonding of Silicon Nitride High-Temperature Co-Fired Ceramic Substrates

**DOI:** 10.3390/ma16072937

**Published:** 2023-04-06

**Authors:** Ling-Feng Wang, Zhe Li, Bo-An Zhou, Yu-Sen Duan, Ning Liu, Jing-Xian Zhang

**Affiliations:** 1 Center of Materials Science and Optoelectronic Engineering, University of Chinese Academy of Sciences, Beijing 100049, China; 2 State Key Laboratory of High Performance Ceramics and Superfine Microstructures, Shanghai Institute of Ceramics, Chinese Academy of Sciences, Shanghai 200050, China; 3 School of Materials and Chemistry, University of Shanghai for Science and Technology, Shanghai 200093, China

**Keywords:** Si_3_N_4_ HTCC, W_2_C, W_5_Si_3_, adhesion strength, interfacial bonding

## Abstract

For the first time, Si_3_N_4_ HTCC has been prepared using W as the metal phase by high-temperature co-firing (1830 °C/600 KPa/2 h) as a potential substrate candidate in electronic applications. It was discovered that the addition of Si_3_N_4_ to the W paste has a significant impact on thermal expansion coefficient matching and dissolution wetting. As the Si_3_N_4_ content increased from 0 to 27.23 vol%, the adhesion strength of W increased continuously from 2.83 kgf/mm^2^ to 7.04 kgf/mm^2^. The interfacial bonding of the Si_3_N_4_ ceramic and the conduction layer was discussed. SEM analysis confirmed that the interface between Si_3_N_4_ and W exhibited an interlocking structure. TEM, HRTEM and XRD indicated the formation of W_2_C and W_5_Si_3_ due to the interface reactions of W with residual carbon and Si_3_N_4_, respectively, which contributed to the reactive wetting and good adhesion strength between the interface. Suitable amounts of Si_3_N_4_ powder and great interfacial bonding were the main reasons for the tough interfacial matching between the Si_3_N_4_ ceramic and the conduction layer.

## 1. Introduction

High-temperature co-fired ceramic (HTCC) is a multilayer ceramic circuit board made from conductive metal paste printed on the ceramic substrate through screen printing according to the circuit design requirements. HTCC is characterized by its high structural strength, good thermal conductivity, excellent chemical stability and high wiring density. Aluminum oxide (Al_2_O_3_) and aluminum nitride (AlN) have been widely used as substrates of HTCC for electronic devices [1,2]. As the most mature ceramic substrate material, Al_2_O_3_ has many advantages as a low-power electronic device substrate material, such as its low dielectric loss, excellent mechanical strength, low preparation cost, etc. [3,4,5,6]. However, the thermal conductivity of Al_2_O_3_ ceramics is too low to meet the heat dissipation requirements for high-power transmission [4]. Metallization based on AlN was carried out in the early 21st century, and now it has been applied in the military and other industries [7,8,9]. The major drawback of AlN is its low mechanical properties and chemical stability [10]. On the other hand, silicon nitride (Si_3_N_4_) possesses high strength [11], toughness [12], heat shock resistance [13], a high dielectric constant [14] and other characteristics, making it a suitable substrate material for high-power and high-density electronic devices [15,16]. In addition, as the substrate of high-power electronic devices, Si_3_N_4_ HTCC not only needs to provide mechanical support and excellent heat dissipation ability but also electrical interconnection for the integrated electronic devices on it. This requires a good combination of the Si_3_N_4_ and the metal circuit, as well as good electrical conductivity of the metal circuit. As the substrate material of HTCC, it has shown broad application prospects in many fields, such as the military industry, communications, environmental protection, aerospace and so on [17].

A Si_3_N_4_ material with high thermal conductivity can be successfully prepared by reaction bonding and post-sintering with the aid of rare earth oxide and alkali metal oxide [18,19]. Therefore, Si_3_N_4_ could be used as the substrate of high-power electronic devices with the solution of metallizing Si_3_N_4_ ceramic. However, there are still few reports on the metallization of Si_3_N_4_ ceramic for applications in electronic devices internally [20,21].

Starting in 1956, La Forge first carried out metallization experiments on Al_2_O_3_ with the activated Mo-Mn method. Then, Reed [22], Fulrath [23] and Twentyma [24] et al. further optimized the experiments. However, the sintering temperature is still too low to densify Si_3_N_4_. The tungsten co-firing method, utilizing tungsten with a high melting point of 3400 °C, is a crucial factor in achieving successful sintering of AlN HTCC above 1700 °C. Song et al. improved the AlN tungsten mentalization and prepared AlN HTCC with an adhesion strength of 2.95 kgf/mm^2^ and a conduction layer resistance of 9.8 mΩ/sq [25]. Hu et al. discussed the role of SiO_2_ in tungsten paste and the electrical and mechanical properties of the substrate. SiO_2_ reacts with Y_2_O_3_, CaO and AlN to form crystalline phases of CaSiAlO and YSiO and vitreous Y-sialon and Ca-sialon at the interface of the substrate and the conduction layer. The conduction layer resistance of the multilayer co-fired substrate is up to 10 mΩ/sq, and the adhesion strength is up to 3.15 kgf/mm^2^ [26]. It was found that Si_3_N_4_ is sintered at temperatures above 1700 °C in the N_2_ atmosphere. As is known to all, Mo reacts with N_2_ at temperatures up to 1500 °C and densifies below 1600 °C by the Mo-Mn method. Consequently, refractory metal W becomes a better choice. As reported in the literature [27,28], the thermal expansion coefficient of Si_3_N_4_ varies from 20 °C to 1000 °C, showing a monotonous upward trend from 1.4 × 10^−6^ K^−1^ to 4.0 × 10^−6^ K^−1^. However, the thermal expansion coefficient of W at 20 °C is ~4.0 × 10^−6^ K^−1^ and increases with rising temperatures. It can exceed 6.0 × 10^−6^ K^−1^ above 1500 °C. Therefore, in order to reduce the thermal stress after sintering, it is necessary to adjust the coefficient of thermal expansion of the conduction layer during sintering by adding Si_3_N_4_ appropriately so as to obtain a good co-firing effect.

In this study, the Si green tape was obtained by tape casting with a suitable addition of Er_2_O_3_ and MgO as sintering additives. The tungsten paste ball milling mixed with Si_3_N_4_ powder was printed on the green tape using screen printing technology. After lamination, hot pressing, debinding, nitriding and sintering, Si_3_N_4_ HTCC with a strong adhesion strength (5.25 kgf/mm^2^) and good film resistance (98.8 ± 20 mΩ/sq) was obtained. The microstructure, phase compositions and mechanism of the interfacial bonding were analyzed.

## 2. Materials and Methods

### 2.1. Raw Materials and Experiment Procedure

Commercial high-purity tungsten powder (W, Innochem, 99.98% metals basis, 1–5 μm, 0.26 wt% O, D50 = 3.47 μm) and Si powder (99.999%, BET 2.205 m^2^/g, 0.42 wt% O, D50 = 5 μm, Haoxi Research Nanomaterials, Inc., Shanghai, China) were used in the present study. High purity Er_2_O_3_ (purity > 99%, Haoxi Research Nanomaterials, Inc., Shanghai, China) and MgO (purity > 99%, Qinhuangdao Yi Nuo Co, Ltd., Qinhuangdao, China, BET 10.448 m^2^/g) were used as sintering additives. Si_3_N_4_ (purity > 99.5%, UBE Industries, Ltd., Kogushi, Japan, 1.26 wt% O, BET 11.6 m^2^/g) was selected as the additive for W paste. The composition of Si slurry with sintering additives was designated as Si:Er_2_O_3_:MgO = 88:9:3 at mass ratio. Polyvinyl butyral (PVB) was used as binder and butyl benzyl phthalate (BBP) was used as plasticizer. W-based screen printing paste with Si_3_N_4_ powders was designated as W:Si_3_N_4_:Er_2_O_3_:MgO = 64–91.23:0–27.23:5.98:2.79 at volume ratio.

The Si, Er_2_O_3_ and MgO powders with defined compositions were dispersed in mixed solvent of ethyl alcohol and xylene at 1:1 (mass ratio) and lab-made dispersant for 24 h. In order to avoid competitive adsorption between binder and dispersant, the slurry with solid loading of approximately 42% was milled after the addition of PVB and BBP for another 36 h. Then, tape casting was carried out on Procast Precision Tape Casting Equipment (Division of International, Ringoes, NJ, USA) at a speed of 120 mm/min with a thickness of 0.4 mm. The obtained green tape was used for the next screen-printing process.

In order to obtain a uniform W screen-printing paste, W, Si_3_N_4_, Er_2_O_3_, MgO and organic vehicle were mixed using a planetary mill for 4 h with WC balls as the medium in polyethylene jars. The organic carrier included ethyl cellulose as binder, span-85 as dispersant, terpineol, butylcarbitol, tributyl citrate, butylbenzyl phthalate as mixed solvent, and hydrogenated castor oil as thixotropic agent. Then, screen printing was performed by screen printer (HP-3050, Haohe Machinery, Dongguan, China) on Si green tape to produce Si/W green tape. Subsequently, after drying at room temperature for 24 h, the Si/W green tape was laminated into uniform 50 mm × 50 mm blocks by laser cutting and hot lamination (9 layers). Then, the laminated green multilayer was heat treated at 200–500 °C with N_2_ as the protective atmosphere to burn out the organics. Dense Si_3_N_4_ HTCC was prepared by reaction bonding and post-sintering method: nitriding at 1420 °C for 2 h under 0.1 MPa N_2_ and sintering at 1830 °C for 1 h 0.6 MPa N_2_ with heating rate of 3 °C/min. Then a slow cooling rate of 3 °C/min to 1500 °C and a second, slower cooling rate of 2 °C/min to 1400 °C with 1 h holding period were adopted.

### 2.2. Characterization

Thermogravimetric analysis of the green body was conducted using TG (STA449C, Netzsch) at a heating rate of 10 °C/min under the flux of air and N_2_ gas at a rate of 30 mL/min. The delta Gibbs energy of the reactions was calculated using HSC Chemistry (HSC Chemistry 9.0). The phase compositions were identified by X-ray diffraction (XRD, D8 Advance, Bruker, Germany) using Cu Kα. The microstructure and element distribution were characterized by scanning electron microscopy (SEM, Magellan 400, FEI Co., Hillsboro, OR, USA), energy dispersion spectroscopy (EDS, Inca of Oxford Instruments, Oxford, UK), transmission electron microscopy and high-resolution transmission electron microscopy (TEM and HRTEM, Tecnai G2 F20, FEI Electron Optics) with energy-dispersive X-ray analysis (EDAX). For TEM samples, thin foils were prepared by focused ion beam technology (FIB, Versa 3D, FEI, Hillsboro, OR, USA). The film resistance was measured using quadrupole probe method (RTS-9, 4probes Tech Ltd., Guangzhou, China).

R = ρ/d(1)

where R (Ω/sq) represents the film resistance; ρ(Ω·m) represents the resistivity; d (m) represents the thickness of the film.

Adhesion strength was tested by vertical tensile method (GB/T 17473.4-2008) by multifunctional tension tester (DAGE4000, DAGE, Aylesbury, UK) on 2 × 2 mm^2^ pad electroplating Ni/Pd/Au.

## 3. Results and Discussion

### 3.1. Sintering Process of Multilayer Green Bodies

**Figure 1:**
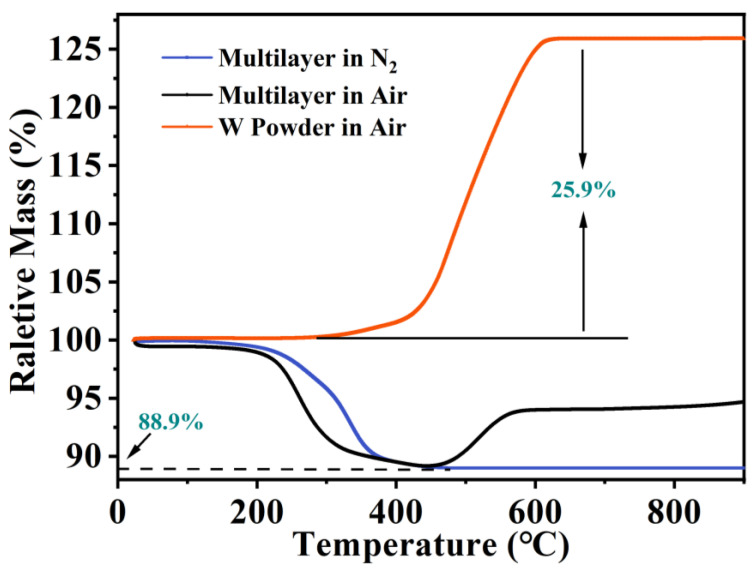
Thermal analysis of green multilayer in nitrogen/air atmosphere and W powder in air.

Figure 1 illustrates the different thermal analysis curves of the green multilayer and W metal powder. The green multilayer was heated from room temperature to 900 °C in an air and N_2_ atmosphere with a heating rate of 10 °C/min. The W powder was heated from room temperature to 900 °C only in air. Prior to nitridation, the organic components in the Si_3_N_4_ multilayer were removed. As Figure 1 shows, tungsten starts to gain weight at 360 °C, and a significant increase of 25.9% is observed after increasing the temperature to 600 °C in air. The thermal analysis curve of multilayer samples in the air also shows an obvious weight gain stage, which starts at 480 °C and ends at 600 °C. This indicates that when the organics are burning in air, the W metal is oxidized, which seriously affects the film resistance performance of the metal circuit [29]. In the N_2_ atmosphere, the thermal analysis curve of the multilayer shows a continuous weight loss of 11.1% from 200 °C to 500 °C. To prevent oxidation of the W layer in air and issues caused by rapid organic volatilization, a debinding process was adopted with a heating rate of 1 °C/min in the nitrogen atmosphere, and multiple holding points were set between 200 and 500 °C.

**Figure 2:**
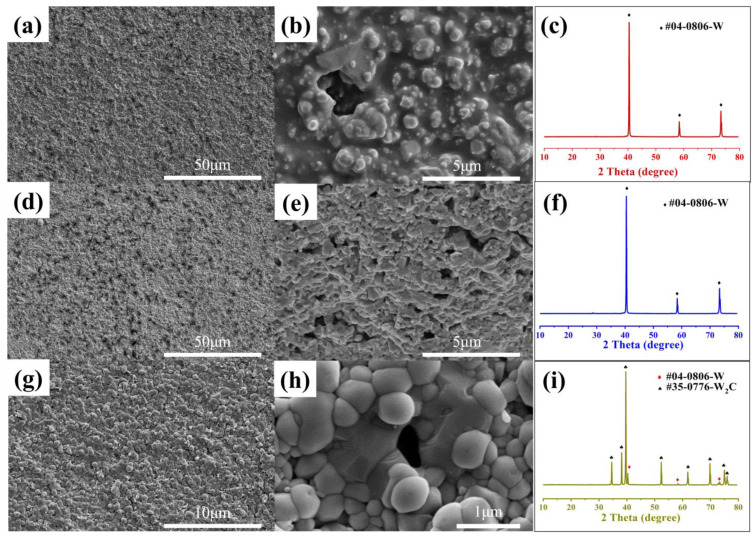
SEM micrographs of the conduction band surface in different stages. (**a**,**b**) Green tape, (**d**,**e**) after debinding, (**g**,**h**) after nitriding with (**c**,**f**,**i**) corresponding XRD pattern.

SEM observations were carried out to observe the surface morphology of the samples at the green tape, after debinding and after nitriding stages. Figure 2 presents the surface morphologies and XRD curves of the samples at these three stages. The surface morphology of the green tape shown in Figure 2a,b clearly displays that W particles were well-coated by the organic film. The chemical composition of the conduction layer, as confirmed by the illustration in Figure 2a, is a W element. After debinding in N_2_, the organic film disappeared, as shown in Figure 2e, and there were interspaces among W particles, as depicted in Figure 2d. The diffraction intensity of W (110) was relatively high and sharpened without any miscellaneous peaks, which indicated that no side effects were observed during the debinding process. After the nitridation step, sphere-like particles with a size of around 1 μm could be seen according to the SEM observation, which is confirmed by Figure 2b,e. Moreover, the conduction layer was preliminarily sintered, and the bonding between W particles was denser, as revealed in Figure 2g,h. The XRD analysis indicated that the W particles on the surface of the conduction layer had obviously changed to tungsten carbide (W_2_C), due to the insufficient pyrolysis of organic compounds in the multilayer during the debinding step in N_2_, and residual carbon (0.7 wt%) was left. Free carbon reacts with tungsten to form W_2_C during nitridation at 1420 °C [30].

2W + C = W_2_C ΔG = −16.97 kJ/mol (2)

### 3.2. Effect of Si_3_N_4_ Additive and Microstructure of Si_3_N_4_ HTCC

Table 1 shows the results of film resistance and adhesion strength testing of Si_3_N_4_ HTCC with varying additions of Si_3_N_4_ powder after sintering. It was observed that the slight addition of Si_3_N_4_ powder had minimal effect on the film resistance of Si_3_N_4_ HTCC, due to its beneficial impact on the densification of the conduction layer, which was a stress-reduced driving process [31]. This resulted in reduced thermal stress during sintering and a slight increase in the film resistance of the conductor layer. Additionally, the adhesion strength of Si_3_N_4_ HTCC was significantly improved with increasing amounts of Si_3_N_4_. The highest adhesion strength of 7.04 kgf/mm^2^ was achieved with an addition of 27.23 vol% Si_3_N_4_, which was more than 3.5 times the standard 2.0 kgf/mm^2^ for electronic devices. This phenomenon could be attributed to two reasons. In one aspect, as shown in Table 1, with the increase in Si_3_N_4_ content, the thermal expansion coefficient of the metal layer at room temperature gradually decreased, approaching that of the Si_3_N_4_ substrate (1.4 × 10^−6^/K at 25 °C). The interface with mismatched thermal expansion coefficients resulted in residual thermal stress during sintering, which affected the strength of the interface bonding [32]. Therefore, the addition of Si_3_N_4_ powder made it closer to that of the Si_3_N_4_ substrate during sintering, reducing the interface thermal stress and thus increasing the bonding strength. In another aspect, dissolution-driven wetting was an important contribution to high-temperature wetting [33]. In this experiment, the Si_3_N_4_ powder was added to the tungsten paste. As the same compound, Si_3_N_4_ powder provided the mutual dissolution and growth of Si_3_N_4_ grains between the conduction layer and Si_3_N_4_ substrate. With the increase in Si_3_N_4_ content, the surface of the metal layer became rougher, providing more active sites for adhesion and contributing to the interlayer adhesion strength. However, the addition of Si_3_N_4_ also resulted in an increase in the film resistance of the conduction layer. Sample 3# exhibited the best performance in terms of both film resistance and adhesion strength. 

**Figure 3:**
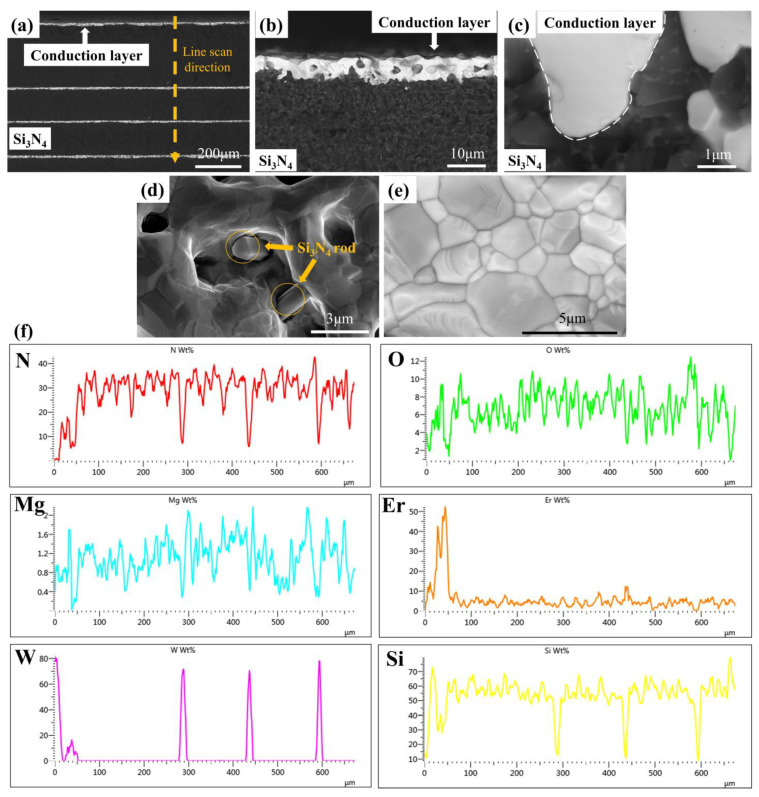
SEM micrographs of Si_3_N_4_ HTCC cross section (**a**) ×120 (**b**) ×2000 (**c**) ×20,000. SEM micrographs of (**d**) exposed Si_3_N_4_ rods in conduction layer (**e**) and its grains after sintering. (**f**) N, O, Mg, Er, W and Si elements’ linear scanning curves of the direction in (**a**).

To gain a more comprehensive understanding of the interfacial bonding of Si_3_N_4_ HTCC, SEM and EDS analyses were conducted on both the cross section and surface of the conduction layer, as shown in Figure 3. Figure 3a displays the cross section of Si_3_N_4_ HTCC, which reveals a tight and alternating distribution of Si_3_N_4_ substrate and conduction layer without any fracture or distortion. The single layer was straight, without fracture or distortion. Figure 3b shows an enlarged cross-section image of the conduction layer surface, which displays a clear boundary, ensuring the uniformity and optimal performance of Si_3_N_4_ HTCC. Moreover, a local amplification (Figure 3c) demonstrates a physical and mechanical interlocking structure between Si_3_N_4_ and the conduction layer, which significantly contributes to the interlayer bonding of Si_3_N_4_ HTCC. Figure 3d shows the morphology of the rod-like β-Si_3_N_4_ embedded in the conduction layer, while Figure 3e presents a local amplification of the sintered conduction layer on the surface, which exhibits well-densified grains. There are also some voids found in Figure 3d. It may be difficult to reach the closest packing of powder after pressureless sintering. Moreover, the conductivity of the metal layer in Si_3_N_4_ HTCC is realized by the contact of W particles after sintering to form a conductive loop. Therefore, as shown in Figure 3b, a clear conductive loop is formed inside the conduction layer, and voids are also found in Figure 3d. However, this will not substantially influence the electric conductivity behavior. Finally, Figure 3f displays the energy spectrum linear scan of Figure 3a. When Er_2_O_3_ and MgO were added to the Si_3_N_4_ substrate as sintering additives, Si, N, Er, O and Mg elements all exhibited the same fluctuation pattern, decreasing sharply when passing through the conduction layer and maintaining a high content when passing through the Si_3_N_4_ layer. Conversely, the W element shows the opposite pattern. It could be found that the densification processes of the Si_3_N_4_ ceramic and conduction layer were both relatively independent and complete.

### 3.3. Discussion of Interfacial Bonding between Si_3_N_4_ Substrate and Conduction Layer

**Figure 4:**
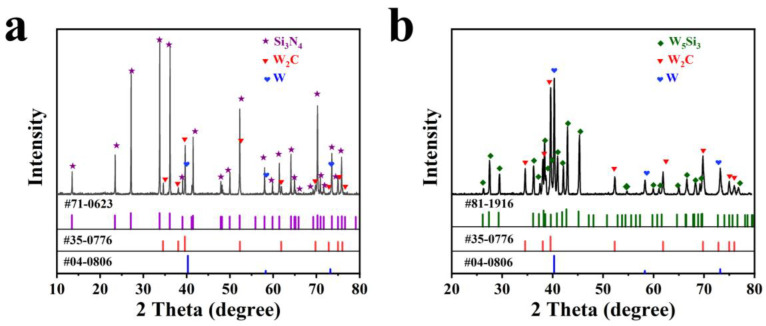
XRD pattern of 3# Si_3_N_4_ HTCC. (**a**) Cross section of Si_3_N_4_ HTCC. (**b**) The surface of conduction layer.

**Figure 5:**
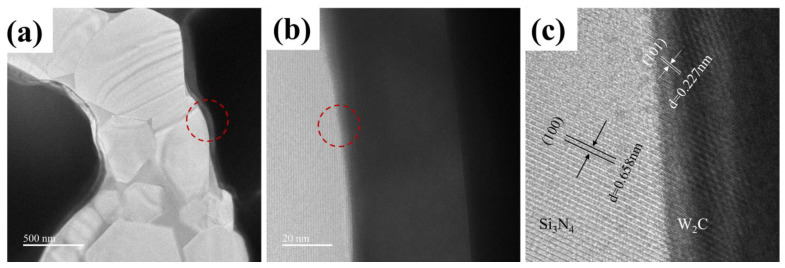
TEM images (**a**) of Si_3_N_4_ grain embedding in conduction layer, (**b**) profile of the interface between Si_3_N_4_ grain and conduction layer pointed by the circle in (**a**,**c**) HRTEM image of the interface pointed by the circle in (**b**).

To investigate the microstructure and composition of the Si_3_N_4_ HTCC, Figure 4 presents the XRD test of the polished cross section and the conduction layer surface for sample 3#. As shown in Figure 4a, the main phase composition of the Si_3_N_4_ HTCC was Si_3_N_4_, W_2_C and W. This supports the view that W reacted with residual carbon after debinding. Figure 4b shows the XRD test of the conduction layer surface, indicating that the crystal phases of the metal layer mainly consisted of W_2_C, W and W_5_Si_3_. To further explore the combination mechanism between the Si_3_N_4_ ceramic and conduction layer, TEM and HRTEM analyses were performed. Figure 5a depicts a Si_3_N_4_ rod penetrating deeply into the conduction layer, thus verifying the physical model of the mechanical interlocking structure. Figure 5b displays the distinct white and gray zones with a clear interface. Figure 5c is a high-resolution image of the selected area in Figure 5b. Two distinct lattice fringes are visible, representing the crystal plane spacing of 0.658 nm of Si_3_N_4_ (100) (PDF: 71-0623) and 0.227 nm of W_2_C (101) (PDF: 35-0776).

**Figure 6:**
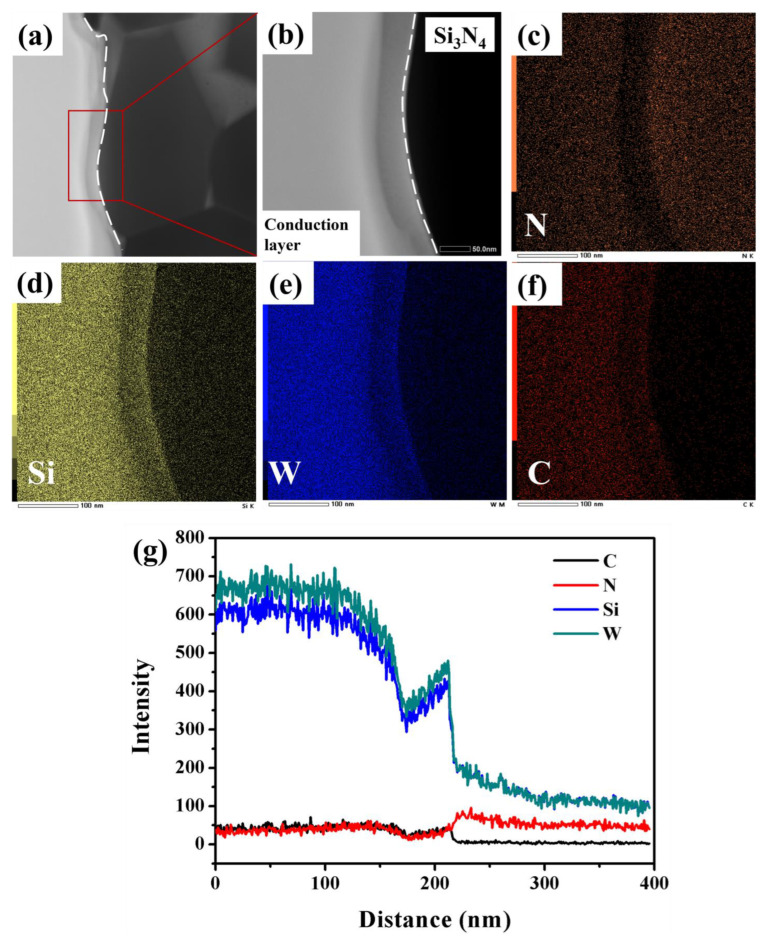
(**a**) Bright-field (BF) image of the interface and (**b**) the local amplification graph for EDS mapping and its corresponding (**c**) N mapping, (**d**) Si mapping, (**e**) W mapping, (**f**) C mapping and (**g**) its line scanning curves.

Figure 6a–g illustrate the EDS analysis results in order to further understand the composition of the interface. EDS mapping clearly shows the distribution of the N, Si, W and C elements in Figure 6c–f, respectively. The change in the energy spectrum of the elements indicates that diffusion occurred during the co-firing process. Prior to co-firing, W was present in the conduction layer, while Si and N were present in Si_3_N_4_. After co-firing, a significant accumulation of Si and N elements was observed on the side of the conduction layer, while the concentration of the W element was lower on the Si_3_N_4_ side. This suggests that the diffusion of N and Si elements to the conduction layer was more pronounced during the co-firing process, while the diffusion of the W element to the Si_3_N_4_ side was relatively weak. Observation of Figure 6g reveals that the degree of aggregation of Si and W elements was relatively consistent with the change in their concentrations. It is clear that Si, W and C elements were enriched in the conduction layer and decreased toward the Si_3_N_4_ substrate. The N element was primarily present in the Si_3_N_4_ substrate and secondarily in the conduction layer. The intermediate platform observed in the EDS mapping was a sample stage due to the difference in thickness from the sample preparation process using focused ion beam (FIB) milling. According to the XRD test, it was determined that the chemical species of W and C in the conduction layer was the W_2_C phase. This indicates that the degree of variation in the concentration of W and C elements is consistent. As Figure 6a indicates, Si_3_N_4_ grains can be clearly observed. Consequently, Si and N elements exist primarily in the form of Si_3_N_4_. However, as it approached the conduction layer, the N element decreased while the Si element increased. Thus, on the conduction layer side, the primary form of the Si element is not Si_3_N_4_ but W_5_Si_3_. According to previous reports and Figure 4b, W reacts with Si_3_N_4_ during sintering as follows [34]:

5W + Si_3_N_4_ = W_5_Si_3_ + 2N_2_ (g) ΔG = −155.62 kJ/mol (1830 °C) (3)

**Figure 7:**
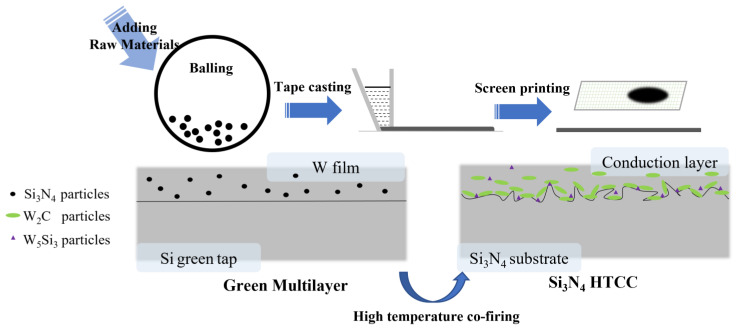
Schematic illustration of the preparation of Si_3_N_4_ HTCC and interfacial bonding mechanism.

Therefore, Si and W elements accumulate only at the interface to form the W_5_Si_3_ compound. The main reason for the accumulation of Si and W on the conduction layer side of the interface could be that the diffusion rate of Si and N atoms was much faster than that of W atoms during co-firing. W_5_Si_3_ is a strong covalent compound, and excessive W_5_Si_3_ would weaken the conductivity of the conduction layer. Moreover, the presence of W_2_C acted as a diffusion barrier for Si_3_N_4_ ceramic [35], which inhibited the interfacial reaction and was beneficial to the conductivity of the conduction layer. According to the line scanning of Si and W elements obtained in Figure 3f, it could be found that the aggregation degree of the Si element dropped sharply as it gradually approached the center of the conduction layer, and the W element appeared to do the opposite. Therefore, it can be inferred that the formation of W_5_Si_3_ was an interfacial reaction, and the degree of reaction decreased rapidly when approaching the middle of the conduction layer. To sum up, the schematic illustration of the preparation of Si_3_N_4_ HTCC and the interfacial bonding mechanism is shown in Figure 7. W_2_C and W_5_Si_3_ coexisted at the interface to provide great reactive wetting. W_5_Si_3_ accumulated at the interface of the conduction layer side and decreased towards the Si_3_N_4_ side and the center of the conduction layer.

## 4. Conclusions

In this study, Si_3_N_4_ HTCC with W paste as the conducting layer was prepared successfully through tape-casting of Si green tape, screen-printing of the W conduction layer and a high-temperature co-firing method. Si_3_N_4_ HTCC with high interfacial bonding (5.25 kgf/mm^2^) and good square resistance (98.8 ± 20 mΩ/sq) can be developed with the addition of Si_3_N_4_ powder.

It was found that the addition of Si_3_N_4_ to the W paste showed an obvious influence on the interfacial bonding. Adding Si_3_N_4_ powder is beneficial to the thermal expansion coefficient matching between the Si_3_N_4_ ceramic and the conduction layer, which leads to less thermal stress and better dissolution-driven wetting.

The interfacial bonding of the Si_3_N_4_ ceramic and the conduction layer was discussed in detail. W combined with residual carbon to form W_2_C and reacted with Si_3_N_4_ to produce W_5_Si_3_, which provided reactive wetting for the interface, and the presence of W_2_C weakened the formation of W_5_Si_3_, which made a balance between great adhesion strength and conductivity for Si_3_N_4_ HTCC. This work provides guidance for promoting the application of the Si_3_N_4_ ceramic in the electronics device field, with a view for it to be an alternate HTCC substrate for AlN in industrial applications.

## Figures and Tables

**Table 1:** The effect of Si_3_N_4_ additive on W film resistance and adhesion strength.

	ω(Si_3_N_4_)/(vol%)	W Film Resistance/(mΩ/sq)	Adhesion Strength/(kgf/mm^2^)	Thermal Expansion Coefficient/(10^−6^/K)
1#	0	110.7	2.83	4.5
2#	8.71	92.08	3.10	4.20
3#	17.42	98.75	5.25	3.91
4#	27.23	146.4	7.04	3.57

## Data Availability

All the data is available within the manuscript.
